# Dataset of ocean vessel traffic in the North Sea

**DOI:** 10.1016/j.dib.2023.109746

**Published:** 2023-10-31

**Authors:** Rory Meyer, Waldo Kleynhans, Marc Portier, Joana Beja, Lennert Tyberghein

**Affiliations:** a Faculty of Engineering, Built Environment and IT, University of Pretoria, Lynnwood Rd, Hatfield, Pretoria 0002, South Africa; b Flanders Marine Institute, InnovOcean Campus, Jacobsenstraat 1, Oostende 8400, Belgium

**Keywords:** AIS (Automated Identification System), Ocean traffic, Heatmap, Vessel activity

## Abstract

Automatic Identification System (AIS) is a technology that allows ships to broadcast their position, course, speed, and other information to other vessels or shore-based stations. By collecting and analysing this data, it is possible to create a heatmap of ship activity in a particular region, such as the North Sea. This heatmap acts as a representation of vessel activity per class. A heatmap in a standard geoinformatics format may be preferable to scientific researchers as it would quickly allow users to overlay their own data onto the vessel density layer thus providing spatial context and an ability to compare their dataset to the distribution and intensity of ship activity in a particular region.

This dataset represents ocean vessel activity in the North Sea for 2022 and was created using AIS data collected using multiple coastal receivers. The dataset was created from reported vessel positions aggregated both spatially and temporally. The end goal of this data processing is to provide a publicly available spatial layer that can be queried to provide monthly vessel traffic statistics for a region in the North Sea.

The data was spatially filtered to only include AIS messages for Latitudes between 49.5 and 53.8 degrees North, and 0.2 and 7 degrees East.

The bounding box was chosen as it includes Belgium canals and the Belgium part of the North Sea.

The dataset has multiple uses as a collaboration dataset, some example of use-cases that this dataset has been used for include using it asa time-series of statistical priors^1^ for vessel classes in order to improve vessel classification algorithms and to visualise vessel behaviour in order to locate potential mooring sites where the risk of potential fishing net snags is low. It has also been used to locate areas of potential anchor scarring in anchorages near ports.

Specifications Table

**Table:** 

Subject:	Maritime Mobility and Transport
Specific subject area:	Maritime domain awareness, human activity monitoring, ship traffic
Data format:	Analyzed
Type of data:	Open Geospatial Consortium (OGC) Web Feature Service (WFS)^a^
Data collection:	AIS data was obtained through the AISHub data sharing network. AISHub aggregates data streams from various sensors. The data was decoded, filtered and inserted into a PostgreSQL database^b^ using open source tools [1]. The sensors used are of various makes and models and there was no ability to determine the source of any specific AIS message. VLIZ installed a COMAR Systems R400N^c^ AIS receiver with VHF whip antenna near the port of Oostende, Belgium. The AIS messages were grouped by vessel ID and class, and ordered in time to create vessel trajectories. These were overlaid onto a pre-generated hexagonal grid in order to calculate the various statistical values used to represent the traffic density.
Data source location:	Primary data source is a database containing AIS messages from ocean vessels carrying AIS transponders, travelling in the Belgium part of the North Sea. This aggregate data product is a secondary dataset derived from the AIS messages. Primary and secondary data is stored and processed at VLIZ: •Institution: Flanders Marine Institute (VLIZ) •City/Town/Region: Oostende, West-Flanders •Country: Belgium •WKT Bounding box of primary and secondary dataset: POLYGON ((0.2 49.5, 7.0 49.5, 7.0 53.8,0.2 53.8, 0.2 49.5))
Data accessibility:	The growing dataset is published in OGC WFS format and hosted on VLIZ GeoServer at https://geo.vliz.be (Layer name= OpenAIS:vessel_density) Repository name: VLIZ Data Centre Data identification number: https://doi.org/10.14284/589 Direct URL to all data (in csv format): https://geo.vliz.be/geoserver/OpenAIS/wfs?service=WFS&version=2.0.0&request=GetFeature&typeName=OpenAIS%3Avessel_density&outputFormat=csv Instructions for accessing these data: Data is made available in OGC WFS format from the VLIZ GeoServer. Users can request the data from the server in any format supported by GeoServer Version 2.23.1 including WMS, WFS, GeoJSON and CSV. The direct URL above is an example on how to pull all available data in CSV format. There are several examples in the Data Description section.

a https://www.ogc.org/standard/wfs/.

b https://www.postgresql.org/.

c https://comarsystems.com/product/r400n-network-ais-receiver-for-coastal-monitoring-applications/.

## 1 Value of the Data

• This dataset is an example of how to aggregate large volumes of AIS data, using open-source tools, into a geospatial product that is publicly available. While there are datasets available that cover the same area, and are released in similar formats [2,3], these datasets have commercial data as an input source. While this data is similar to the EMODnet Human Activities Vessel Density dataset,^2^ it used open-source software and open datasets to create a data product that could easily be recreated for areas not covered but the EMODnet layer and allows users to compare multiple overlapping datasets to determine accuracy and suitability for their purposes.
• This dataset uses OGC standards to allow researchers to easily compare their own spatial datasets with one that describes human activity in the Belgium part of the North Sea. Some specific examples include comparing GPS bird tracks to fishing vessels, comparing vessel traffic to undersea acoustic sensor networks, and comparing vessel anchorages with benthic floor scarring.
• Subsets of this dataset can be requested for specific months or vessel classes, allowing users to use it to map changes in vessel traffic over time or to map class specific locations like fishing grounds or anchorages.
• Using this dataset can allow users to highlight areas of high traffic that may have an increased risk of vessels collision [4].
• Vessel traffic is a major contributor to air pollution and a map showing the location, time spent within an area and the class of vessels within that area can assist with modeling air pollution and underwater acoustic noise pollution [5]. The grid is also hexagonal which approximates the propagation of pollution better than a standard rectangular grid.
• This dataset can be used as an input feature in an AIS vessel classification algorithm [6]. It allows users to retrieve statistical priors for specific regions which would provide more context to unlabeled vessel detections and could improve classifier accuracy.

## 2 Data Description

**Fig. 1:**
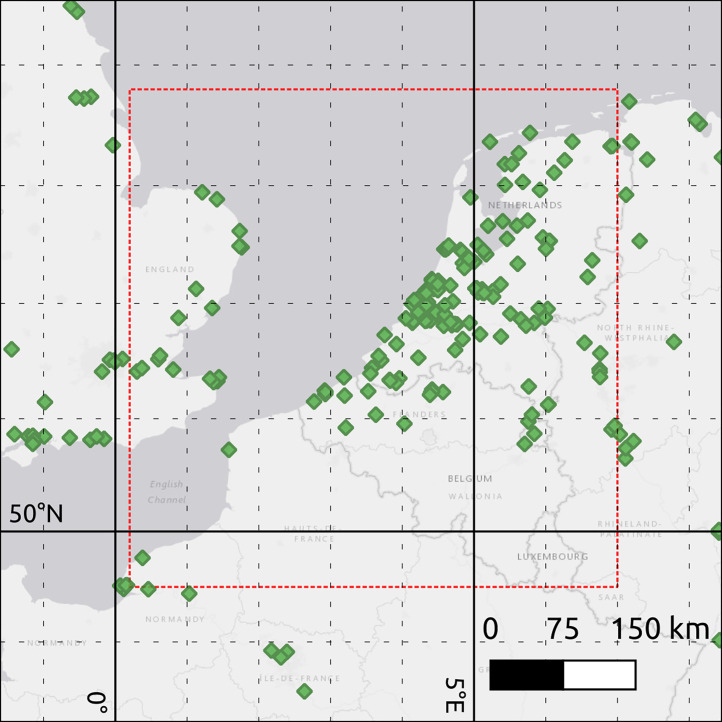
Bounding box (red dashes) for AIS filter. Green diamonds show locations of AISHub receiver stations.

The data is a geographic representation of vessel traffic and is accessible by a geographical data server; GeoServer. GeoServer allows users to filter and request the underlying data in many different formats or spatial projections.The bounding box of the data is shown below in Fig. 1.

GeoServer allows for both Machine to Machine communication and user interaction for data querying/download. Users can constrain the URL above by using other information, e.g. month, year, type, thereby extracting only the information they are interested in, e.g. constrain to month='May' and (vessel) type='Cargo' and year=2023

https://geo.vliz.be/geoserver/OpenAIS/wfs?service=WFS&version=1.1.0&request=getFeature&typeName=OpenAIS:vessel_density&cql_filter=month='Jan'%20AND%20type='Cargo'%20AND%20year=2023&outputFormat=csv

**Fig. 2:**
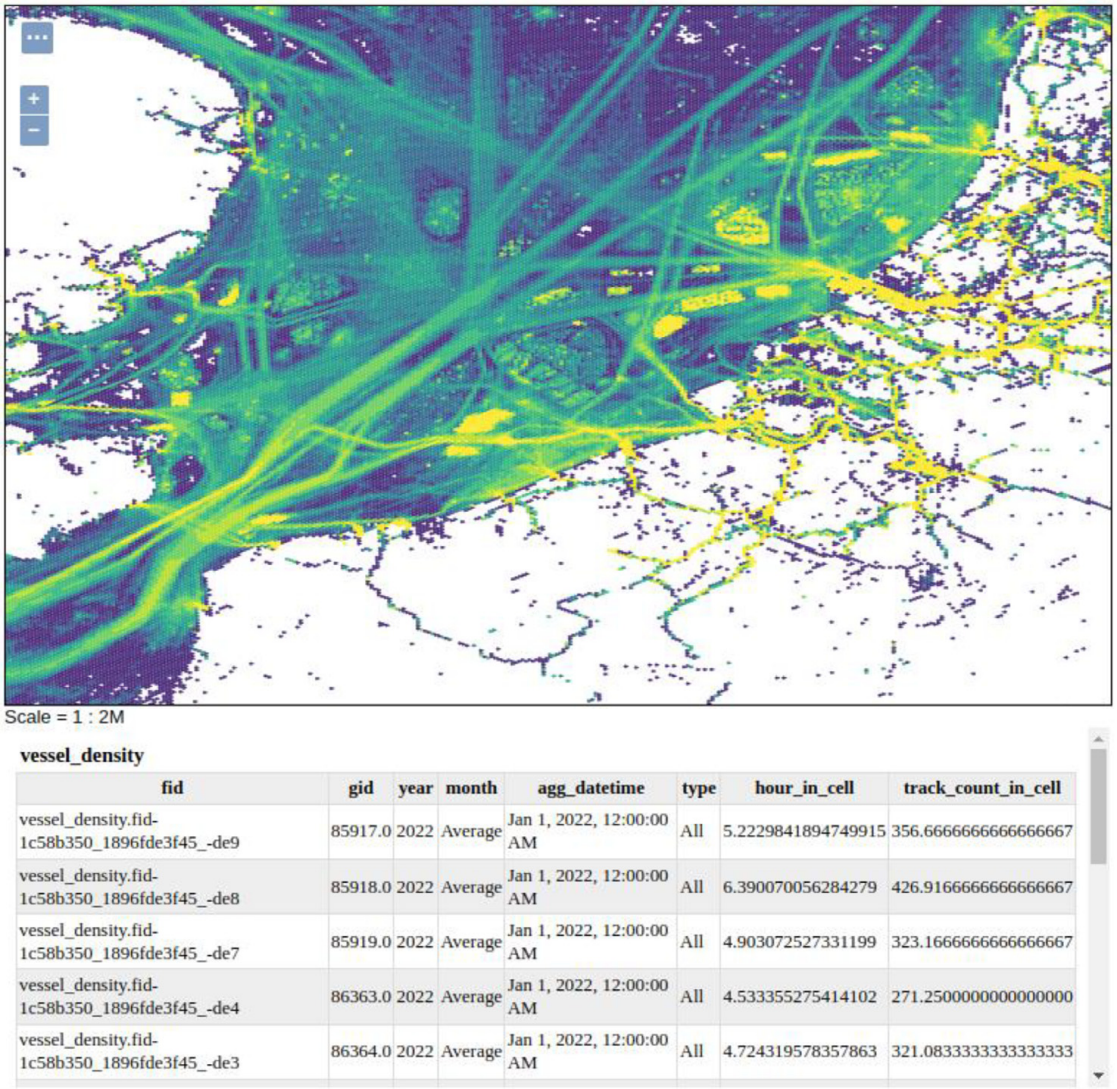
Example of the data being retrieved as a web map with table describing a subset of data selected by a user.

Fig. 2 shows the data represented by a web map and a table showing data for grid cells near the mouse click.The URL used to retrieve this map is

https://geo.vliz.be/geoserver/OpenAIS/wms?service=WMS&version=1.1.0&request=GetMap&layers=OpenAIS%3Avessel_density&bbox=2.0%2C51.4%2C4.2%2C52.7&width=768&height=485&srs=EPSG%3A4326&styles=&format=application/openlayers

While the data can be shown or downloaded in various formats the data can have the fields shown in Table 1. If the dataset were to be downloaded as a CSV file the dataset would be represented by a table with the columns shown in Table 1.

**Table 1:** Example data row for single grid cell.

Column Name	Description	Example
**FID**	Geoserver generated Field ID (FID). This is a unique ID for each database row.	vessel_density.fid–5c9490ad_186938bf9cc_-2202
**Gid**	Geometry ID (GID). This is a unique ID for each cell geometry. This is repeated multiple times in the database for different vessel classes, months etc.	117620
**Year**	The year that the data was captured in. YYYY format.	2022
**Month**	A three letter representation of the month the data was aggregated over.	Nov
**agg_datetime**	The ISO datetime that the data was aggregated over. This is the first day of each month that the data represents and is included to allow the data to be ordered in time.	2022-11-01T00:00:00
**Type**	The reported AIS vessel class.	Other
**hour_in_cell**	The total number of hours that the vessels spent within the hexagon grid, grouped by date and vessel class	0.54286331
**track_count_in_cell**	The number of unique tracks that intersect with the grid geometry, grouped by date and vessel class	36
**Geom**	Definition of hexagon boundaries. Stored in Well-Known-Binary format in the database but can be converted to WKT or some other geometry format by Geoserver. The geometry is in the WGS84 projection.	POLYGON ((3.71894290337161 52.04212138184412, ... 3.71894290337161 52.04212138184412))

## 3 Experimental Design, Materials and Methods

### 3.1 Raw data

Streaming AIS data is obtained from AISHub.^3^ The data is collected from multiple coastal AIS receivers but without a station ID attached, making it difficult to understand which stations are active and inactive.

The raw data is streamed to the VLIZ server at about 500 hundred messages per second and is then reduced to around 120 messages per second after spatial filtering.

**Fig. 3:**
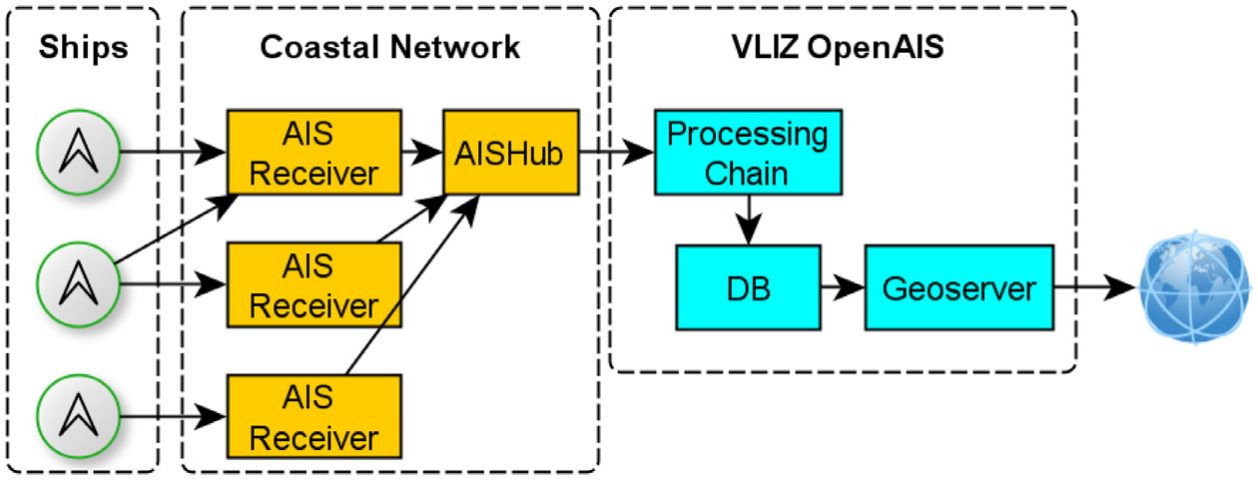
AIS propagation block diagram.

The data streaming in real time from AISHub is decoded, filtered and stored into the Postgres+TimescaleDB+PostGIS database using open-source AIS tools.^4^ Fig. 3 shows a block diagram describing how vessel location data is moved from on board AIS transmitters to the publicly available dataset through the VLIZ OpenAIS instance.

### 3.2 Data preprocessing

The raw AIS is first decoded into a dictionary of data with fields dependent on what AIS message type was received. The decoder used is “libais” available from PyPi.^5^ After decoding the messages that have spatial data, mainly position report messages, are then filtered to remove messages outside the area of interest. Not all messages contain spatial information that can be limited to the North Sea and these are stored without filtering.

Messages are then grouped into two categories: position reports and voyage reports. In some cases a single message can contain enough information to be used in both categories. The position reports are inserted into a position report table in the database and the same is done for voyage reports.

### 3.3 Data processing

Once the data is stored in the database more complex aggregations are possible. This dataset is one such example. The data is aggregated over a 1 km2 hexagonal grid in order to create a value for the number of hours per month that each (and all) vessel type(s) spend within the grid cell. A hexagonal grid was chosen for several reasons:

• Underwater sound propagation is radial in nature and hexagonal grids approximate the propagation pattern better than square grids do
• Determining traffic routing is better when the distance between edges and neighbours are equal. Diagonal routing with square grids is problematic.

The aggregate is calculated by creating a moving window over the AIS data where segments are created from AIS messages and the prior AIS message for each vessel. Data checks are done on all segments and those with a length of 0 (due to duplicate messages) or greater than 0.05 degrees (approximately 5 km) are discarded. The 0.05 degree limit is defined in order to remove large jumps in vessel positions that might cut across land or rivers and be unrepresentative of normal ship behavior. The time differences from consecutive AIS messages are added together for each grid cell and vessel class. For segments that cross multiple grid cells the time is split between grid cells based on the portion of the segment within the cell over the total segment length.

A simplified example is shown below. In this example 3 messages are received from a ship exiting the port of Oostend. The 3 messages are placed into 2 segments with lengths L1 and L2. The time delta of the first segment, t1 - t0, is associated with the grid cell Grid_n_ while the second segment is associated with both cell Grid_n_ and Grid_m_. The time delta is split between the two cells, by calculating the portion of the segment within each grid cell and apportioning the time delta to the cell relative to the size of the length within the cell. The assumption that segment length is proportional to time spent in a cell breaks down when there are large changes in vessel velocity in a segment. Fortunately the AIS protocol states that large changes in a ship's velocity require more frequent AIS message transmissions.

The methods used to calculate the values for each cell were chosen to remove known issues with the AIS protocol like irregular transmission rates, poor reception far from shore, and duplicate messages from receivers located near each other. Some other heatmap products [[2], [7]] use AIS message point locations as a proxy for density but this can lead to underestimating density in locations where messages might not be received due to poor receiver coverage. The method chosen here aggregates the line segments created by a time series of messages from vessels  and has several advantages over normal point based heatmap aggregations [8]**.**

**Fig. 4:**
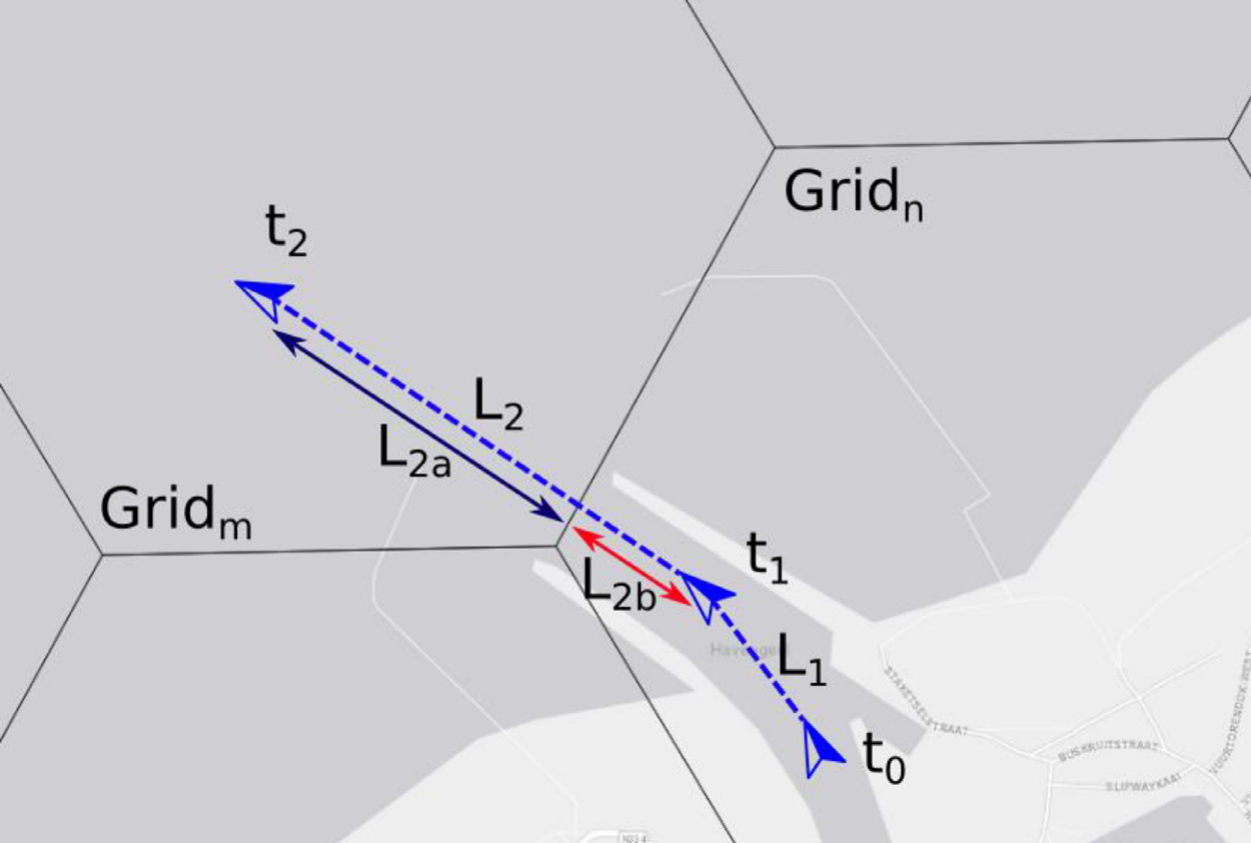
Simplified example of time aggregation for a vessel crossing multiple grid cells.

As shown in Fig. 4 the time associated with Grid_n_ is:

Tn=(t1−t0)+(t2−t1)*(L2b/L2)

These aggregations are calculated each day and then averaged into a monthly aggregate at the end of each month.

The vessel class associated with AIS messages received is derived from the “voyage reports” types of AIS messages. This has several classes for vessels as described in Table 2.

**Table 2:** Mapping of AIS class codes to published dataset classes.^a^

AIS Class Code	Vessel Class	Description
0	Not Available	No description available
1 – 19	Reserved	Reserved
20 – 29	Wing in ground	Wing in ground (WIG), all ships of this type
30	Fishing	Fishing
31	Towing	Towing
32	Large Towing	Towing: length exceeds 200m or breadth exceeds 25m
33	Dredging	Dredging or underwater ops
34	Diving	Diving ops
35	Military	Military ops
36	Sailing	Sailing
37	Pleasure Craft	Pleasure Craft
38,39	Reserved	Reserved
40 – 49	High Speed Craft	High speed craft (HSC), all ships of this type
50	Pilot	Pilot Vessel
51	S&R	Search and Rescue vessel
52	Tug	Tug
53	Port Tender	Port Tender
54	Anti-pollution equipment	Anti-pollution equipment
55	Law Enforcement	Law Enforcement
56, 57	Spare	Spare - Local Vessel
58	Medical Transport	Medical Transport
59	Non-combat	Noncombatant ship according to RR Resolution No. 18
60 – 69	Passenger	Passenger, all ships of this type
70 – 79	Cargo	Cargo, all ships of this type
80 – 89	Tanker	Tanker, all ships of this type
All Others	Other	Other Type, all ships of this type
	All	Sum of all ship types

a Taken from the description of the AIS protocol here: https://gpsd.gitlab.io/gpsd/AIVDM.html.

### 3.4 Validation

The data heatmap was compared to the EMODnet Human Activities Vessel Density layer [2] for 2022 for the same region. The comparison was done both spatially and statistically to determine any errors and their location. It must be noted that this layer uses a rectangular grid in the ETRS89-LAEA^6^ projection while the OpenAIS vessel density layer is hexagonal and, while created with a Belgium Lambert projection (EPSG SRID: 31370^7^), is stored in EPSG: 3857.^8^ These differences result in non-overlapping grid cells that will have a significant impact on areas with tight shipping lanes like anchorages, rivers and ports.

**Fig. 5:**
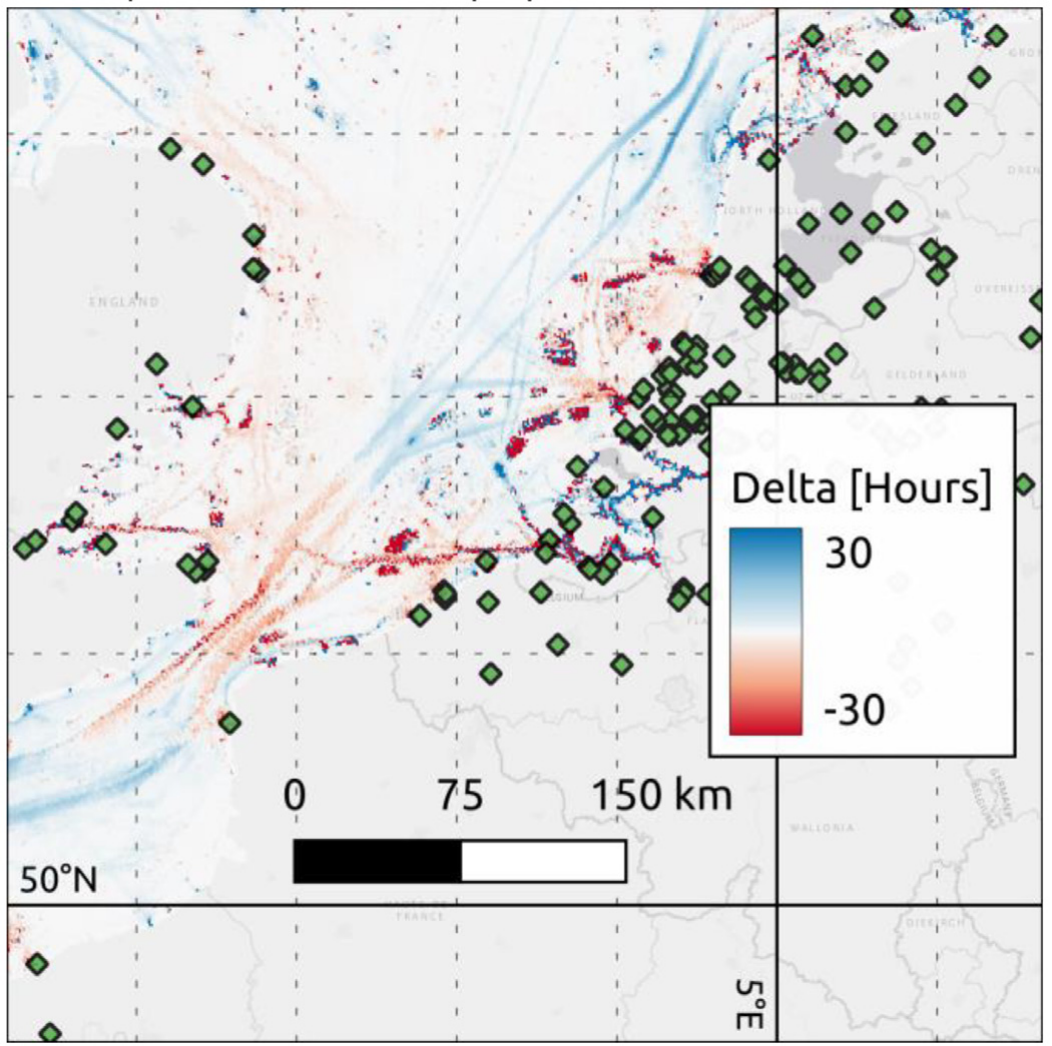
EMODnet vessel density layer - OpenAIS vessel density layer for 2022. Green diamonds represent AISHub receiver stations.

Fig. 5 shows the difference between the 2022 EMODnet Human Activities Vessel Density layer and the 2022 OpenAIS Vessel Density layer.

Areas shown as blue in the map are areas where the EMODnet Vessel Density layer is reporting higher vessel density than the OpenAIS layer, while red is the opposite. The majority of differences occur in regions of the ocean far offshore or where there are few coastal receivers. There are also significant, sharp, differences in regions where vessel density is tightly grouped, like ports, anchorages and canals. The differences in the tightly grouped regions can partly be attributed to differences in the grids chosen, hexagonal vs rectangular, but this does not explain all the differences noted.

**Fig. 6:**
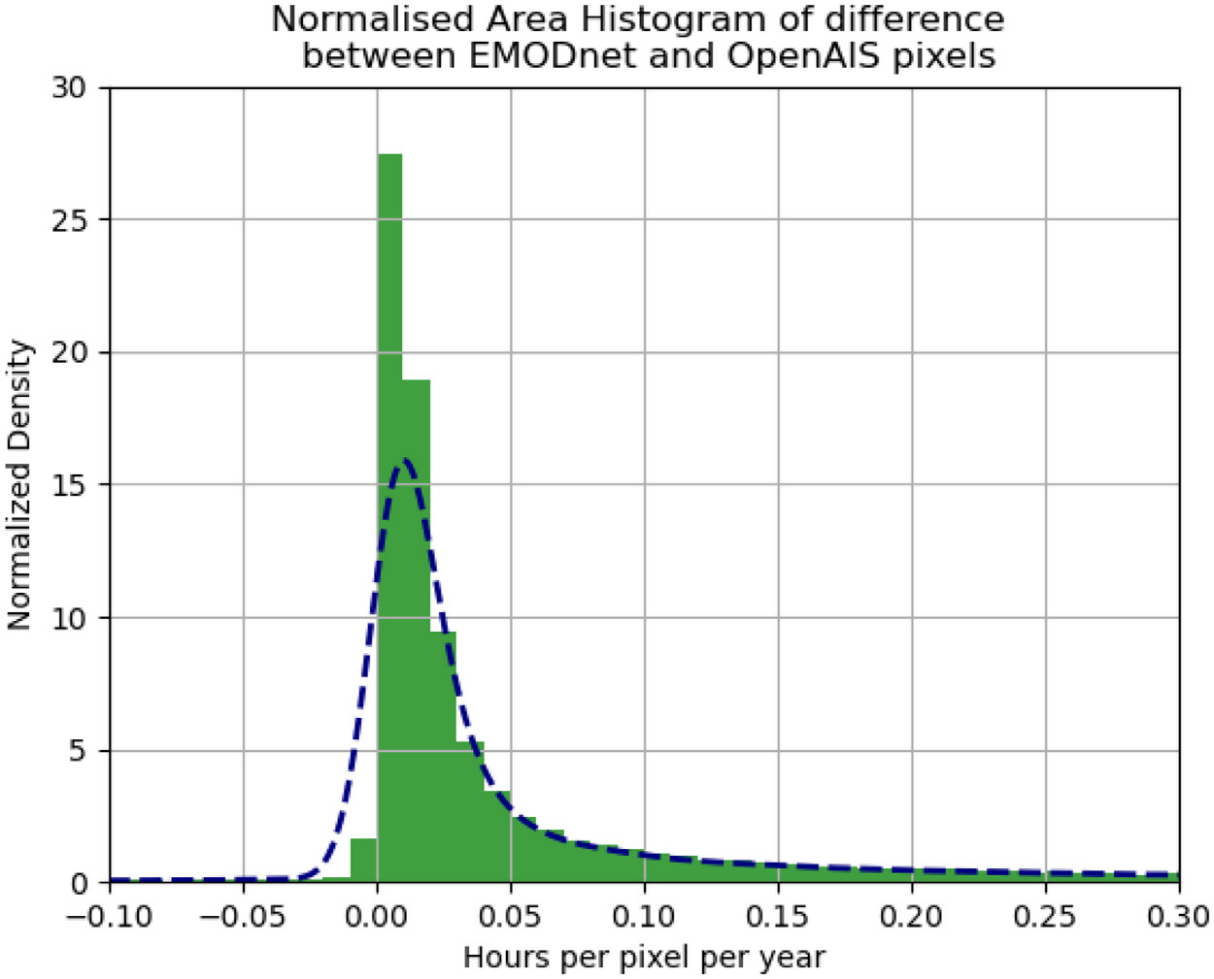
Histogram of pixel differences between EMODnet and OpenAIS.

Fig. 6 shows a histogram of the differences for all overlapping pixels:

The histogram is slightly tilted towards the positive half indicating that EMODnet typically has a higher pixel value than OpenAIS on average but is also centred around zero indicating a general agreement between the two density layers.

## 4 Limitations

The limited amount and range of coastal AIS receivers create several limitations. Vessels beyond the reception range of coastal receivers, or in areas without coastal receivers are not detected. Vessels with class B transceivers are also not detected as well as Class A transceivers as they have a lower RF output power. Class B transceivers are typically carried by smaller vessels like sail boats, fishing and recreational vessels.

There are also several computational limitations on the VLIZ server infrastructure which sometimes can result in timeouts when attempting to visualise the data as a web map. There are plans in place to address the computational limitations but will require time.

## Ethics Statement

The primary AIS data used in this dataset complies with the data providers terms of use, specifically; “Every AISHub contributor is allowed to use the data for free”.

This work meets the requirements for ethical publishing (https://www.elsevier.com/authors/policies-and-guidelines). The work does not include chemicals, procedures or equipment that have any unusual hazards inherent in their use, nor does it involve the use of animal or human subjects. No studies on patients or volunteers have been performed.

## CRediT authorship contribution statement

**Rory Meyer:** Conceptualization, Methodology, Software, Writing – original draft. **Waldo Kleynhans:** Supervision, Writing – review & editing. **Marc Portier:** Project administration. **Joana Beja:** Data curation, Writing – review & editing. **Lennert Tyberghein:** Resources, Funding acquisition.

## Conflict of Interest

The authors declare that they have no known competing financial interests or personal relationships that could have appeared to influence the work reported in this paper.

## Data Availability

OpenAIS:vessel_density (Original data) (VLIZ Geoserver)

## Appendix Supplementary materials

Image, application 1

## Footnotes

2 https://emodnet.ec.europa.eu/geonetwork/srv/eng/catalog.search#/metadata/0f2f3ff1-30ef-49e1-96e7-8ca78d58a07c.

## Footnotes

3 https://www.aishub.net/.

4 https://open-ais.org/.

5 https://pypi.org/project/libais/.

6 https://epsg.io/3035.

7 https://epsg.io/31370.

8 https://epsg.io/3857.
